# The impact of management traps on surgical strategies in parathyroid benign and malignant tumors-related PHPT: a retrospective cohort study

**DOI:** 10.3389/fonc.2025.1535089

**Published:** 2025-05-15

**Authors:** Guang-wen Zhu, Xue Lv, Zhan Jiao

**Affiliations:** ^1^ Thyroid Team, First Affiliated Hospital, Dalian Medical University, Dalian, China; ^2^ Department of Nuclear Medicine, First Affiliated Hospital, Dalian Medical University, Dalian, China; ^3^ Department of Surgery, 967 Hospital of the Joint Logistics Support Force of PLA, Dalian, China

**Keywords:** primary hyperparathyroidism, parathyroid adenoma, atypical parathyroid tumor, parathyroid carcinoma, surgical strategies, management trap

## Abstract

**Background:**

Reducing the incidence rate of persistent/recurrent HPT after surgery is the key to the treatment of PHPT. The pitfalls of preoperative, intraoperative, and postoperative management in PHPT patients and their potential impact on surgical strategies need to be comprehensively investigated.

**Methods:**

The demographic, biochemical, radiological results and other clinical data of the enrolled 112 patients with primary hyperparathyroidism undergoing surgical treatment were obtained from our database in this retrospective cohort study. One-way analysis of variance was used for normally distributed variables, and Kruskal-Wallis H test was used for non-normally distributed variables. Pearson’s chi-square test or Fisher’s exact test was used for categorical variables, as appropriate.

**Results:**

The patients were divided into parathyroid adenoma group and atypical parathyroid tumor + parathyroid carcinoma group. The serum calcium levels, serum PTH levels in the APT+PC group were higher than those with benign lesions, but there was some overlap; and the clinical data showed no specificity in the differentiation of benign and malignant parathyroid tumors. A more significant finding in this cohort was that the tumor size was significantly larger in persistent/recurrent HPT group than in non-persistent/recurrent group (30.0 ± 12.6 mm vs.19.1± 8.3 mm, p < 0.01).

**Conclusion:**

In PHPT, there are pitfalls in preoperative, intraoperative, and postoperative management of parathyroid tumors, which affect the choice of surgical strategies. It is prudent to utilize the tumor-free margin En bloc resection in a variety of parathyroid neoplasms, in order to seek the chance of cure and avoid reoperation as much as possible.

## Introduction

Primary hyperparathyroidism (PHPT) is a typical manifestation of parathyroid benign and malignant tumors, becoming the third most common endocrine disease with an incidence rate of about 27.7 per 100,000 person-years (1), in which distant metastatic or recurrent disease can lead to death due to uncontrolled severe hypercalcemia. Parathyroid carcinoma is a refractory malignant malignancy. inadequate and non-radical surgery is the strongest prognostic factor for recurrence and mortality (2).

A growing body of evidence supports that the pitfalls in the management of parathyroid tumors in primary hyperparathyroidism (PHPT) have a significant impact on patient outcomes. For example, preoperative imaging limitations, intraoperative under-recognition, postoperative surveillance gaps, etc. Despite accumulating rich experience in the management of PHTP, varying management approaches have been employed over time, but consensus has not yet been reached in certain aspects, and most studies only focus on the management of parathyroid adenoma or parathyroid carcinoma separately, which seems to be out of the clinical situation. Here, we focus on the pitfalls of managing parathyroid benign and malignant tumors related-PHPT and their potential impact on treatment strategies.

## Materials and methods

This retrospective cohort study included 112 patients with PHPT who were consecutively diagnosed and treated between November 2016 and November 2023 at a regional tertiary medical institution. All cases met the PHPT criteria (serum calcium was elevated, unsuppressed serum parathyroid hormone (PTH) level, and there were no other causes of hypercalcemia) and were confirmed by histopathology after parathyroidectomy according to the new 2022 World Health Organization classification criteria (3). The diagnosis of parathyroid carcinoma (PC) requires one of the following histologic features: ① vascular invasion, ② lymphatic invasion, ③ perineural invasion, ④ invasion into adjacent anatomic structures, and ⑤ documentation of metastatic disease (4). Atypical parathyroid tumor (APT) demonstrates atypical features that are worrisome for PC, but do not show unequivocal invasion as would be required for diagnosis of PC, lacks unequivocal capsular, vascular, or perineural invasion or invasion into adjacent structures or metastases (3). Persistent/recurrent hyperparathyroidism (HPT) was defined as persistent or recurrent disease (hypercalcemia with high or inappropriately normal serum PTH level) after 6 months of parathyroidectomy.

The preoperative, intraoperative and postoperative clinical data were retrospectively obtained from our medical database. These included demographics, clinical manifestations (such as bone involvement, urinary system damage, gastrointestinal symptoms, gallbladder calcification, and hypercalcemic crisis), biochemical indices, ultrasound, 99mTc-MIBI SPECT/CT, ^18^F-FDG PET/CT, other radiological findings, surgical notes, pathology, follow-up and clinical outcomes. This retrospective study was conducted in accordance with the principles of the Helsinki Declaration and was exempted by the Ethics Committee of the institution.

### Statistical analysis

SPSS Statistics 22.0 (Chicago, Illinois, USA) was used for statistical analysis. After normality testing, the categorical variables were expressed as percentages, the normally distributed variables were expressed as mean ± standard deviation (SD), and the non normally distributed variables were expressed as median and 25th and 75th interquartile ranges (Q25, Q75). One-way analysis of variance was used for normally distributed variables, and Kruskal-Wallis H test was used for non-normally distributed variables. Pearson’s chi-square test or Fisher’s exact test was used for categorical variables, as appropriate. Two-sided P < 0 .05 was considered statistically significant.

## Results

**Figure 1:**
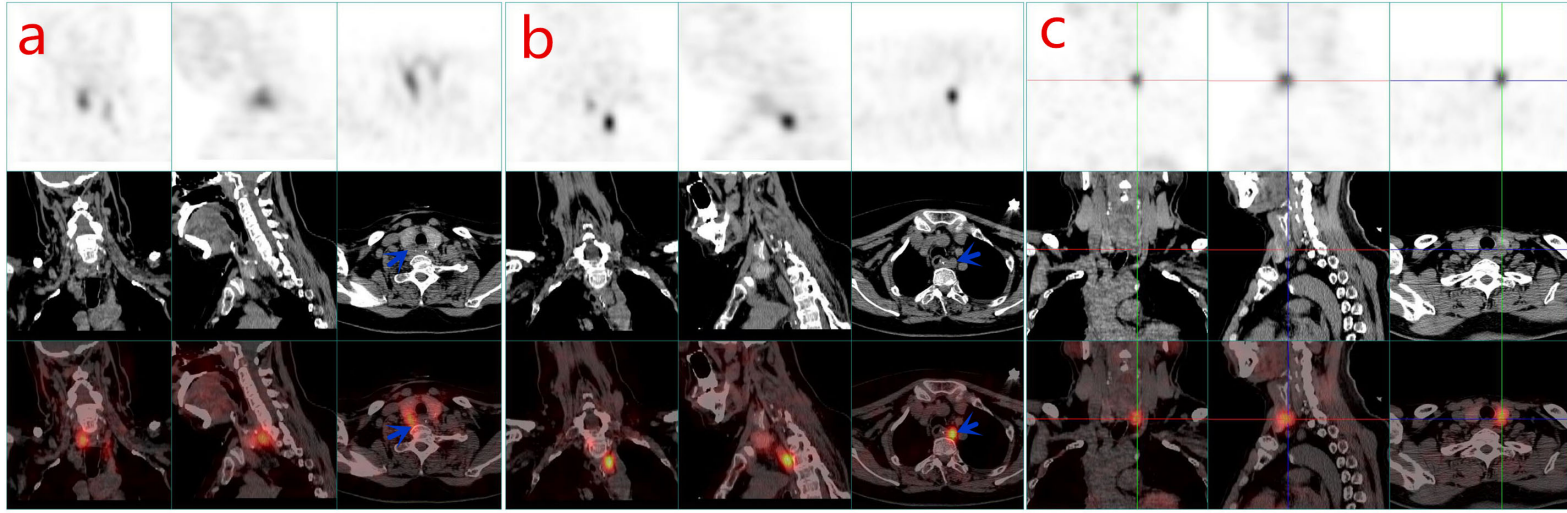
Functional nuclear medicine imaging with single photon emission CT (SPECT) combined with anatomical X-CT (SPECT/CT) has improved the sensitivity and specificity for many clinical applications. 99mTc-MIBI SPECT/CT is the first-line imaging modality for detecting parathyroid tumors in PHPT. Local tomography and image fusion techniques significantly improve the positive rate of detection. The first row is SPECT image, the second row is X-ray CT image, and the third row is SPECT and CT fused image obtained by the hybrid system. 99mTc-MIBI SPECT/CT: In the right parathyroid bed region **(a)** and the upper mediastinum **(b)**, two lesions with high 99mTc-MIBI uptake were clearly shown in the transverse, coronal and sagittal planes (blue arrow). *In situ* and ectopic parathyroid adenomas were confirmed by postoperative pathology. PET/CT is the second-line imaging modality for detecting parathyroid tumors in PHPT. The first row is PET image, the second row is X-ray CT image, and the third row is PET and CT fused image obtained by the hybrid system. 18F-FDG PET/CT: In the left parathyroid gland bed region **(c)**, a high 18F-fluorodeoxyglucose metabolic lesion was clearly displayed in the transverse, coronal and sagittal planes. Parathyroid adenoma was confirmed by postoperative pathology.

A total of 112 patients were enrolled in this study, 104 cases with parathyroid adenoma (PA), 6 cases with APT, 2 cases with PC. The structural features of APT share some similarities with PC, such as adhesion to adjacent structures but without invasion, nuclear atypia, and the presence of thick fibrous bands within the tumor, and even Ki-67 proliferation index can be greater than 5% (4); therefore, ATP can be in the same group as PC. The patients were divided into PA group and APT+PC group in our cohort. The demographics, biochemical indicators and radiological results were shown in **Table 1**. After at least 6 months of follow-up, there were 21 cases with persistent/recurrent HPT and 83 cases with non-persistent/recurrent HPT, and their clinical characteristics were listed in **Table 2**. Two patients with PA were localized by 18F-FDG PET/CT and a patient with *in situ* PA and ectopic PA clearly localized by 99mTc-MIBI SPECT/CT (**Figure 1**). In this queue, no cancer features such as firm, adherent, or invasive parathyroid nodules were observed during surgery in two PC patients.

**Table 1:** Clinical features of patients with parathyroid adenoma, atypical parathyroid tumor and parathyroid carcinoma confirmed by surgical pathology.

Characteristic	Overall	PA	APT+PC	χ^2^ Z, t	p-value
No. of patients, n(%)	112	104 (92.9)	8 (7.1)		
Age(yr)	55.7 ± 15.8	53.8 ± 14.8	56.2 ± 13.4	-0.867	*0.421*
Sex				0.159	0.653
Female, n(%)	82 (73.2)	77 (74.0)	5 (62.5)		
Male, n(%)	30 (26.8)	27 (26.0)	3 (37.5)		
Preoperative outcomes					
PTH (pg/mL)	264.7 (142.6~769.4)	199.4 (129.2~386.1)	924.0 (306.4~1041.1)	-2.808	0.005*
Calcium (mg/dL)	2.9 ± 1.0	2.8 ± 0.4	3.1 ± 0.6	-75.936	0.000*
Ultrasound,n(%)	112 (100.0)	77/104 (74.0)	6/8 (75.0)	0.159	*0.653*
MIBI-SPECT/CT, n(%)	50 (44.6)	37/42 (88.1)	7/8 (87.5)	0.000	*1.000*

PA, parathyroid adenoma; APT, atypical parathyroid tumor; PC, parathyroid carcinoma; PHPT, primary hyperparathyroidism; PTH, parathyroid hormone; MIBI-SPECT/CT, 99mTc-MIBI-SPECT/CT; *, *p <0.05.*

**Table 2:** Clinical features of patients with persistent/recurrent vs non- persistent/recurrent.

Characteristic	Overall	Persistent/Recurrent	Non-P/R	χ2 Z, t	p-value
No. of patients, n(%)	104	21 (20.2)	83 (79.8)		
Age(yr)	55.8 ± 11.1	57.6 ± 10.6	55.3 ± 11.2	-0.816	*0.417*
Sex				0.258	*0.611*
Female, n(%)	75 (72.1)	15 (71.4)	60 (72.3)		
Male, n(%)	29 (27.9)	6 (28.6)	23 (27.7)		
Preoperative outcomes					
PTH (pg/mL)	213.0 (12.2~429.6)	270.8 (125.7~735.6)	204.2(127.2~392.0)	-0.660	*0.509*
Calcium (mg/dL)	2.9 ± 0.3	2.9 ± 0.3	2.8± 0.3	-0.871	*0.386*
Tumor-size (mm)	21.3 ± 10.2	30.0± 12.6	19.1± 8.3	-3.696	0.001*

P/R, persistent/recurrent; PTH, parathyroid hormone; Persistent/recurrent HPT was defined as persistent or recurrent disease 6 months after parathyroidectomy. *, *p* <0.05.

No specific clinical manifestations were found to accurately distinguish PC or APT from PA. The serum calcium levels, serum PTH levels in the APT+PC group were higher than those in benign lesions, but there was some overlap. A more significant finding in this cohort was that the tumor size was significantly larger in the persistent/recurrent HPT group than in the non-persistent/recurrent HPT group (30.0 ± 12.6 mm vs.19.1± 8.3 mm, *p* < 0.01).

Due to the limited sample size, a multivariable logistic regression analysis assessing potential risk factors was deemed statistically underpowered and therefore not pursued. This restriction may affect the generalizability of observed associations.

## Discussion

The most common cause of PHPT is a single PA, accounting for approximately 80%-85% (5). A multicenter retrospective analysis of the incidence rate of parathyroid tumors showed that APT accounted for about 2.8% of parathyroidectomies. PC is an extremely rare malignant tumor, accounting for about 1% of PHPT (6). In our cohort, PC accounted for 1.8% of all sporadic PHPT cases in a regional tertiary hospital in China.

There are many pitfalls in the management of parathyroid tumors, firstly due to limitations in preoperative imaging and laboratory results. The only definitive management for PHPT is surgery. However, the preoperative differential diagnosis of PA, APT, and PC is full of challenges. Although the APC+PC subgroup in this article has a relatively small sample size, previous literature and this study indicated that the serum calcium levels, serum PTH levels in the APT+PC group were higher than those in benign lesions, but there was some overlap (7). Clinical data from PHPT suggest that there were no specific differences in symptom characteristics between PA, APT, and PC. Also, the ultrasound, 99mTc-MIBI-SPECT/CT and 18F-FDG PET/CT results showed that they were also insufficient to distinguish between PA, APT and PC. Furthermore, due to the ineffectiveness of cytology in distinguishing benign and malignant parathyroid tumors, as well as the risk of tumor cell seeding and spreading, Fine-needle aspiration (FNA) is not recommended before surgery (8). This also results in the limited availability of genetic testing and specific immunohistochemical markers for parathyroid tumors by FNA biopsy prior to surgery.

Secondly, the management pitfalls of parathyroid tumors also include intraoperative under-recognition, postoperative surveillance gaps, etc. In the intraoperative and even post-operative settings, regardless of preoperative suspicion of PC, a firm, adherent, and aggressive parathyroid mass was observed intra-operatively, usually suspected to be PC. However, in this cohort, two patients with PC did not have the above characteristics, and it has been reported that up to one-third of PCs macroscopically appeared to be benign lesions, which did not show adhesions (9). Therefore, the possibility of PC cannot be ruled out based on the macroscopic morphological characteristics alone (10) and it is also important to understand that frozen-section analysis cannot be used to distinguish malignant from benign disease; the diagnosis of parathyroid carcinoma is often based on postoperative histopathology (11).

PA, APT and PC all contribute to persistent/recurrent HPT disease. The general consensus is that the only definitive treatment for PHPT is surgery, which may lead to permanent cure. However, once the initial surgery fails, the consequences mainly include the occurrence of persistent/recurrent HPT. The reported incidence of persistent/recurrent HPT after surgery varied widely. Other consequences include the likelihood of parathyromatosis, poor clinical outcomes, a shorter median disease-free period, and a reduced likelihood of long-term cure after PC reoperation. Notably, parathyromatosis may occur in the setting of primary hyperparathyroidism because of local persistent/recurrence disease due to intraoperative rupture of the capsule of a benign parathyroid lesion (12), which highlights the necessity for complete resection of the parathyroid tumor and avoidance of intraoperative rupture. In fact, there were few records of tumor tissue spillage during surgery. In this cohort, the tumor size in the persistent/recurrent HPT group was significantly larger than that in the non-persistent/recurrent group, which may be related to the greater likelihood of the adequacy of resection or intraoperative rupture in larger tumors.

There was 8% evidence of local recurrence after En bloc resection, while it was 51% after standard parathyroidectomy (13). However, only in cases of intraoperative suspicion of PC, En bloc resection with avoidance of capsule rupture is strongly recommended by the American Association of Endocrine Surgeons Guidelines (8). Notably, previous reports have shown that only about 12.5% of case series receive En bloc resection (14). Indeed, to date, it remains impractical to distinguish PC or APT from PA before surgery, and even there are intraoperative and postoperative conflations and pitfalls. In addition, the positive surgical margin of PC lead to worse overall survival (15). Thus, in the setting of patients with PHPT, regardless of what kind of surgical procedures related to parathyroid gland surgery, the tumor-free margin En bloc resection for parathyroid tumors avoiding capsular disruption is appropriate. This regimen may lead to a reduction in persistent/recurrent HPT and better outcomes, which may provide a clear reason to recommend this treatment option in patients with PHPT, especially in PC related cases. Although it has been reported that more extensive surgery for parathyroid cancer did not result in better overall survival rate (11), a positive pathological margin generally indicates a higher rate of recurrence and the need for reoperation. Of course, En Bloc resection for all parathyroid tumors may be associated with a higher risk of unnecessary complications, and the surgeon should balance the benefits of the patient against the surgical risks, adopt the appropriate surgical procedures; Especially for larger parathyroid tumors, such as those larger than 3.0 cm, cystic parathyroid adenomas, or ectopic lesions, an experienced surgeon is required to perform the En Bloc resection in order to maximize patient benefits and avoid unnecessary complications.

The management of parathyroid tumors has changed significantly with advances in diagnostic imaging, surgical techniques, and molecular understanding. However, there are several areas that warrant further investigation, including preoperative diagnostic biomarkers, novel imaging tracers, intraoperative molecular markers, genomic classifiers, AI-based imaging to enhance risk stratification and personalized precision therapy.

The study has several limitations. First, this is a single-center retrospective study, which inevitably has a small sample size and may have some biases and sampling variation, Secondly, there are few medical records of tumor tissue overflow during surgery. In our opinion, this is also the limitation of this study. However, given this comprehensive discussion of the preoperative, intraoperative and postoperative conflations and pitfalls of parathyroid tumors, these results remain valuable to clinicians and researchers.

In conclusion, current evidences have demonstrated that there are pitfalls in the preoperative, intraoperative, and postoperative management of parathyroid tumors in PHPT. Actually, it is prudent to utilize the tumor-free margin En bloc resection in a variety of parathyroid neoplasms, in order to seek the chance of cure and avoid reoperation as much as possible. Of course, if the exact diagnosis is PC, then extended En bloc resection should be appropriate, including the ipsilateral thyroid lobe and tissue resection, even the involved recurrent laryngeal nerve to obtain negative margins.

## Data Availability

The original contributions presented in the study are included in the article/supplementary material. Further inquiries can be directed to the corresponding author.
